# Epigenetic Mechanisms in Type 2 Diabetes Retinopathy: A Systematic Review

**DOI:** 10.3390/ijms221910502

**Published:** 2021-09-28

**Authors:** Agostino Milluzzo, Andrea Maugeri, Martina Barchitta, Laura Sciacca, Antonella Agodi

**Affiliations:** 1 Department of Clinical and Experimental Medicine, Endocrinology Section, University of Catania Medical School, 95122 Catania, Italy; agostino.milluzzo@unict.it (A.M.); lsciacca@unict.it (L.S.); 2 Department of Medical and Surgical Sciences and Advanced Technologies “GF Ingrassia”, University of Catania, Via S. Sofia 87, 95123 Catania, Italy; andrea.maugeri@unict.it (A.M.); martina.barchitta@unict.it (M.B.)

**Keywords:** retinopathy, type 2 diabetes, diabetes complications, epigenetic, miRNA, lnc-RNA, DNA methylation

## Abstract

Diabetic retinopathy (DR) is one of the main causes of vision loss in middle-aged economically active people. Modifiable (i.e., hyperglycaemia, hypertension, hyperlipidaemia, obesity, and cigarette smoke) and non-modifiable factors (i.e., duration of diabetes, puberty, pregnancy and genetic susceptibility) are involved in the development of DR. Epigenetic mechanisms, modulating the oxidative stress, inflammation, apoptosis, and aging, could influence the course of DR. Herein, we conducted a systematic review of observational studies investigating how epigenetics affects type 2 diabetes retinopathy (T2DR). A total of 23 epidemiological studies were included: 14 studies focused on miRNA, 4 studies on lnc-RNA, one study on both miRNA and lnc-RNA, and 4 studies on global or gene-specific DNA methylation. A direct relation between the dysregulation of miR-21, miR-93, and miR-221 and FPG, HbA1c, and HOMA-IR was identified. A panel of three miRNAs (hsa-let-7a-5p, hsa-miR-novel-chr5_15976, and hsa-miR-28-3p) demonstrated a good sensitivity and specificity for predicting T2DR. Little evidence is available regarding the possible role of the long non-coding *MALAT1* dysregulation and *MTHFR* gene promoter hypermethylation. Despite these initial, encouraging findings potentially suggesting a role of epigenetics in T2DR, the use in clinical practice for the diagnosis and staging of this complication encounters several difficulties and further targeted investigations are still necessary.

## 1. Introduction

Diabetic retinopathy (DR) is a specific microvascular complication of diabetes mellitus (DM) which results in the damage of small blood vessels and neurons of the retina. It is one of the leading causes of vision loss in middle-aged economically active people, accounting for 4.8% of the number of cases of blindness (37 million) worldwide [1]. It is worth underlining that, with the increasing incidence of DM, the number of people DR has been estimated to rise to 191 million by 2030 [2]. A European multicentre study reported that DR prevalence among patients with type 1 diabetes (T1D) ranges from 25% to 60% [3]. The burden of DR appears to be lower in type 2 diabetes (T2D) patients, with a prevalence that ranges from 25% in United Kingdom to 40% in Italy [4,5,6]. The risk factors of DR can be broadly classified into modifiable (i.e., hyperglycaemia, hypertension, hyperlipidaemia, obesity, and cigarette smoke) and non-modifiable factors (i.e., duration of diabetes, puberty, pregnancy and genetic susceptibility). These risk factors are also involved in the development of both diabetic nephropathy, neuropathy and macrovascular complications [7]. Recent strides in comprehension and awareness of risk factors for DR significantly improved its prevention and the management of DR patients. Specifically, the increasing access to community screening programs has led to a decline in the prevalence and incidence of DR, especially in developed countries [8]. Moreover, several randomised controlled trials have shown that early treatment of DR might reduce the risk of severe visual loss by 57% [9]. However, emerging evidence also suggests that a complex gene–environment interaction is involved in the pathogenesis of diabetes-related microvascular complications [10]. Epigenetic mechanisms—including DNA methylation, histone modifications, and miRNAs and long non-coding RNA (lnc-RNA) regulation—contribute to the dysregulation of signalling pathways involved in oxidative stress, inflammation, apoptosis, and aging, and modulate the expression of several key genes in DM [11,12]. Both preclinical and clinical studies in diabetic patients provided strong evidence concerning the contribution of histone modifications, post-transcriptional RNA regulation, and DNA methylation in diabetes-related microvascular complications by regulating molecular pathways involved in the pathogenesis of these complications. Here, we conducted a systematic review to summarise current evidence from observational studies investigating the relationship between DR and epigenetic mechanisms.

## 2. Materials and Methods

The present systematic review was carried out in accordance with the Preferred Reporting Items for Systematic Reviews and Meta-analyses (PRISMA) statements [13] and the Cochrane Handbook’s guidelines [14].

### 2.1. Literature Search

Epidemiological studies evaluating the association between DR and epigenetic mechanisms were systematically searched on PubMed-Medline (PubMed.gov: available online: https://pubmed.ncbi.nlm.nih.gov/, accessed on 1 June 2020), and Web of Science databases (https://www.webofscience.com/wos/woscc/basic-search, accessed on 1 June 2020), from inception to June 2020. The search strategy applied the following combination of Mesh terms: (“MicroRNAs” [Mesh] OR “DNA Methylation” [Mesh]) AND “Diabetic Retinopathy” [Mesh]. Reference lists of potentially eligible articles were also screened.

### 2.2. Selection Criteria

The following inclusion criteria must have been satisfied: (1) epidemiological observational studies; (2) evaluating the association of DR; (3) with epigenetic mechanisms (i.e., DNA methylation and miRNA or lnc-RNA expression); (4) in patients with T1D or T2D. Only studies published in peer-reviewed journals were included, with no limitations with regard to publication date or language. Conversely, previous systematic reviews and meta-analyses, commentary articles, and editorials were excluded.

### 2.3. Data Extraction and Quality Assessment

Titles and abstracts of all identified articles were independently reviewed by two authors, applying the selection criteria described above. The full texts of all eligible articles were further reviewed to assess whether selection criteria were fully met. Controversies were resolved by consultation with a third author for obtaining consensus. The following information was extracted by two investigators: first author, study year, location, study design, sample size, type of DM, level of DNA methylation and/or miRNA/lnc-RNA expression, main findings.

## 3. Results

### 3.1. Characteristics of the Included Studies

**Figure 1:**
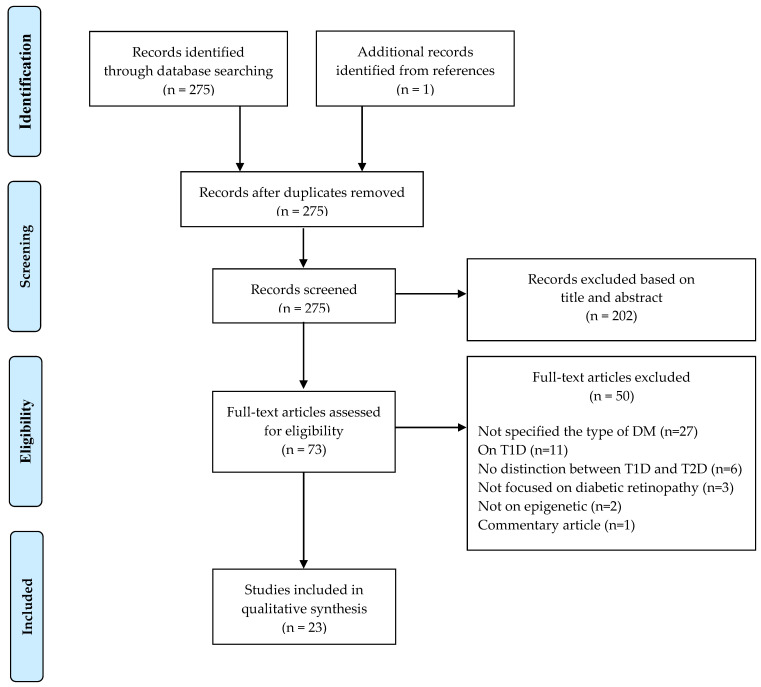
PRISMA 2009 flow diagram of study selection.

Figure 1 illustrates the literature selection process. A total of 275 articles were screened once duplicates had been removed. According to selection criteria, 202 articles were excluded after reading article title and abstract: of these, 129 were not conducted on human, 72 were literature reviews, meta-analyses or microarray/network analyses, and one study did not focus on DR. Out of the 73 articles that underwent the full-text screening, 50 were excluded for the reasons detailed in Figure 1; therefore, 23 epidemiological studies investigating the role of epigenetic in the pathogenesis of T2D retinopathy (T2DR) were included in the systematic review (Figure 1).

Regarding the epigenetic biomarkers, 14 studies investigated the expression of miRNA in plasma (*n* = 6), serum (*n* = 6), aqueous humour (*n* = 1), or tears (*n* = 1) samples. Another four studies investigated the plasma (*n* = 3) or serum (*n* = 1) level of different lnc-RNA, while one study focused on both miRNA and lnc-RNA serum expression. The global or gene-specific DNA methylation was evaluated in four studies.

The majority of the included studies compared the level of the investigated epigenetic biomarkers in T2D patients also affected by DR with a group without DR and a healthy control group (HC) (*n* = 17). Among them, one study also enrolled a group of patients affected by impaired glucose tolerance (IGT). Instead, in six studies the control group was represented only by T2D patients without DR (*n* = 5) or healthy subjects (*n* = 1). No studies examined histone modifications.

### 3.2. miRNA Profiling

In the last decade, several investigations have been carried out to study the influence of epigenetic on T2DR. Most of these studies focused on the analysis of miRNA expression in different human samples, mostly plasma or serum (Table 1). MiR-93, miR-126, and mirR-221 have been studied in cohorts of both T2D and T1D patients [15,16,17,18,19,20].

Mir-93 influences the progression of DR by regulating angiogenesis, although the exact mechanism remains unclear [33,34]. In a cohort of 140 T2D patients, Zou and colleagues found a higher expression of plasma miR-93 and VEGF in the group with DR compared to the group with no ocular complications [15]. Therefore, the expression of miR-93 was directly associated with course of disease, the level of HbA1c and FPG. More recently, a study including patients with both type 1 and type 2 diabetes, confirmed an up-regulation of miR-93 in patients with DR, in particular with in those affected by severe forms and recurrent vitreous haemorrhages [18].

Confirming the role of miRNA dysregulation in the pathogenesis of DR, Liu and colleagues observed a significant overexpression of serum miR-221, VEGF, and angiotensin II (Ang-II) in T2D patients compared to healthy subjects and a progressive up-regulation in diabetic patients without DR, non-proliferative diabetic retinopathy (NPDR), proliferative diabetic retinopathy (PDR) [17]. In addition, the level of miR-221 was positively related to HbA1c, HOMA-IR, VEGF, and Ang-II. In vitro observations, performed using human umbilical vein endothelial cells (HUVECs), showed that a hyperglycaemic environment up-regulates the expression of miR-221 inducing retinal cells proliferation, migration, apoptosis, vascular endothelial hyperplasia, ischemia and neovascularisation [35]. These data suggest the potentially utility of miR-221 in predicting both the occurrence and progression of DR. Similar results were obtained by García de la Torre and colleagues in T1D patients [19]. They found an increased level of miR-221 in endothelial progenitor cells (EPCs) of T1D patients compared to HC. Moreover, T1D patients with DR had higher expression of miR-221 than those without DR.

In the study by Rezk and colleagues, the reduced expression of miR-126 was related to the onset of both macro-vascular complications and DR [16]. A similar reduction of miR-126 expression was also observed in the large cohort of the EURODIAB study among T1D patients with diabetes-related vascular complications, particularly with PDR [20]. MiR-126 is highly represented in endothelial cells playing a crucial role in endothelial homeostasis and angiogenesis influencing VEGF signalling by blocking two negative regulators of the VEGF pathway, Sprouty-related protein (SPRED1) and phosphoinositol-3 kinase regulatory subunit 2 (PIK3R2/p85-b) [16,36].

In 2017, Jiang and colleagues evaluated plasma expression of miR-21 in T2D patients with different severity of DR, showing a positive relation between miR-21 expression and the course of retinopathy [21]. In particular, they observed a significant increase of miR-21 level in the PDR group compared to both the NPDR and control groups. These data suggest the influence of miR-21 as an indicator for the severity of T2DR. Although the authors demonstrated that the role of miR-21 in the pathogenesis of DR was related to T2D course, HbA1c, fasting plasma glucose, and the homeostasis model assessment of insulin resistance (HOMA-IR), further investigations are necessary to clarify the molecular mechanisms determining the involvement of miR-21 on T2DR. In a previous study, the aberrant expression of miR-21 in aqueous humour samples of diabetic patients was associated with retinal fibrosis mediated by the transforming growth factor beta (TGF-β) [37]. Moreover, a possible angiogenetic role of miR-21 could be speculated: Liu and colleagues found that miR-21 induces vascular endothelial growth factor (VEGF)-mediated tumour angiogenesis through targeting phosphatase and tensin homolog (PTEN) and activation of AKT/ERK signalling [38,39].

A very interesting study by Kamalden and colleagues demonstrated that miR-15a was increased in the plasma of T2D patients with DR, although a correlation with DR grade was not found [22]. Moreover, the authors observed a similar expression of miR-15a in the human Müller cells (MIO-M1), human retinal endothelial cells (HRECs), human retinal pigment epithelial cells (HRPEs), and rat Müller cells (rMC-1) exposed to high glucose or advanced glycated end-product environment, thus excluding a role of hyperglycaemia. Notably, by culturing rat pancreatic β-cells (INS-1) cells in high-glucose media, they demonstrated an exosome transfer of miR-15a from pancreatic to retinal cells. These findings support the finding that miR-15a, produced in pancreatic β-cells, is transported in blood exosomes towards retinal cells, where could promote DR, increasing oxidative stress. In a previous study, Hirota and colleagues observed an increased expression of miR-15a in aqueous humour of diabetic patients with PDR [33]. It has been shown that miR-15a controls the insulin synthesis in pancreatic β-cells [40]. In T2D, the increased insulin production due to insulin-resistance, could led to an increase of miR-15 synthesis. MiR-15a is also known to regulate angiogenesis by suppressing fibroblast growth factor (FGF2) and VEGF [33].

Conflicting data are available regarding the role of miR-423, miR-29b, and miR-200b on the course of DR. Mir-423 seems to be inversely related with DR. Blum and colleagues measured the plasma level of miR-423 in T2D patient with different retinal conditions and healthy subjects, showing an overall not statistically significant negative trend in correlation to diabetic retinopathy progression [23]. In contrast, other studies, carried out in patients with different types of diabetes, reported an increased expression of miR-423 in diabetic patients affected by retinopathy [33,41]. These studies speculated on a possible cross talk between miR-423 and VEGF signalling and NOS function influencing miR-423 influencing vascular retinal proliferation [23]. However, further investigations are necessary to clarify these findings.

An inverse relation with T2DR was also observed for the plasma level of miR-29b and, most of all, miR-200b [24]. Furthermore, in another study, Zeng and colleagues observed a higher expression of Mir-29b in diabetic patients with DR. The authors identified the dysregulation of miR-29b-3p/SIRT1 as a potential mechanism of human retinal microvascular endothelial cells (HRMECs) apoptosis in DR [42].

In a recent study, three miRNAs (hsa-let-7a-5p, hsa-miR-novel-chr5_15976, and hsa-miR-28-3p) were shown to be significantly associated with DR of patients with T2D [25]. This panel presented a sensitivity and specificity of 0.92 and 0.94, respectively, for predicting DR and 0.93 sensitivity and 0.86 specificity for differentiating early stage NPDR from late-stage PDR, representing a potential diagnostic biomarker for DR. The authors, studying the in vitro proliferation rates of HRMECs with overexpression of hsa-let-7a-5p, described an increased proliferation of these cells demonstrating how miRNAs may be involved in the pathogenesis of DR.

Yang and colleagues analysed the expression of miR-155, regulatory T (Treg) cells (CD4+ CD25+ Foxp3+), and TGF-β in peripheral blood of T2D patients with different severity of DR in comparison with T2D patients not affected by DR and HC [26]. MiR-155 is a multifunctional miRNA essential to the immune response by regulating the Treg cells cytokines secretion [43]. This study observed a significantly higher expression of miR-155 and a significantly lower percentage of Treg cells in the PDR and background diabetic retinopathy (BDR) groups compared to not diabetic retinopathy (NDR) group and in the PDR group compared to the BDR group. These data suggest a possible role of miR-155 in the pathogenesis of T2DR by regulating the Treg cells with TGF-β.

Additionally, miR-122 seemed to be related with the severity of DR. A prospective study by Pastukh and colleagues showed that the serum expression of miR-122 increased from HC to NDR group and from NDR to NPDR groups [27]. Instead, the miR-122 was significantly reduced in patients with PDR. Notably, a positive trend was observed between miR-122 levels and the number of serum endothelial progenitor cells. The increase of miR-122 in patients with DR is explained by its role in preventing angiogenesis and proliferation, while the decline in patients with more severe grade of DR may represent an inhibition or exhaustion of the anti-angiogenic anti-proliferative defence system.

Another miRNA influencing the onset and progression of DR is miR-20b. Recently, Shaker and colleagues observed a significant down-regulation of miR-20b in T2D patients with progressive severity of DR [28]. The authors indicated this miRNA as promising novel biomarkers for prediction of DR severity, distinguishing PDR from NPDR. Nevertheless, these findings should be confirmed by larger studies and the pathogenesis of miRNAs involvement in DR should be clarified.

The role of miR-31, miR-3939, miR-1910-3p was investigated in different serum samples of different cohorts of T2D patients [29,30]. Nevertheless, these studies excluded their involvement in the development of T2DR.

While most of the studies analysed the expression of miRNA in plasma or serum samples of T2D patients, several studies have investigated these epigenetic biomarkers in vitreous humour or tears [31,32]. In 2019, Chen and colleagues explored the miRNA and piwi-interacting RNA (piRNA) profile in the aqueous humour of nine PDR and nine cataract control patients [31]. In addition, a mice retinopathy model supported the investigation of the relation between miRNA expression and angiogenetic processes. Among the eight miRNAs and thirty piRNAs analysed, the relative expression patterns of miR-93-5p (confirmed in the mice model), -184 and -150-5p in aqueous humour were differently expressed in patients with PDR compared to cataract controls, suggesting a role in the in the pathogenesis of PDR in T2D. This differential expression of miRNA was predicted to regulate Rho protein signal transduction, neurotransmitter uptake and histone lysine methylation.

A case–control study by Pinazo-Durán and colleagues studied the RNA concentration and the miRNAs expression in tears samples of 77 T2D patients and 55 healthy subjects. A significant difference in both total RNA and miRNAs concentration between T2D and HC groups was observed. Moreover, the authors found a direct correlation between a panel of 14 miRNAs and age, obesity, T2D duration, and a negative correlation with visual acuity. Nevertheless, other studies are necessary to better investigate the role of miRNA expression in tears as molecular biomarkers for DR.

### 3.3. lnc-RNA Profiling

The need to improve the knowledge about the molecular basis of DR has directed, in recent years, the efforts of researchers toward the identification of novel molecular biomarkers involved in the pathogenesis of this condition. Lnc-RNA is a functional non-protein-coding RNA of at least 200 nucleotides which modulating both transcriptional and post-transcriptional regulation plays pivotal functions in several human diseases [44]. However, very little evidence is currently available regarding the influence of lnc-RNA on DR (Table 2).

Recently, Shaker and colleagues evaluated the serum expression of homebox antisense intergenic RNA (HOTAIR) and metastasis-associated lung adenocarcinoma transcript 1 (MALAT1) in T2D patients with various degree of DR in comparison to those not affected by this complication [28]. A significant increase in HOTAIR and MALAT1 was observed in both NPDR and PDR groups compared to the NDR group, indicating a possible role of HOTAIR and MALAT1 as promising novel biomarkers for prediction DR. In contrast with these results, Toraih and colleagues observed a reduced expression of circulating MALAT1—also called nuclear-enriched abundant transcript 2 (NEAT2)—and retinal non-coding RNA2 (RNCR2) in T2D patients with DR compared to those without DR [45]. Nevertheless, the expression of MALAT1 and RNCR2 did not correlate with the severity of retinopathy and with the response to aflibercept therapy [45].

Two different studies evaluated the expression of MALAT1 in aqueous humour of diabetic patients [48,49]. In both of them, the level of MALAT1 was significantly increased in patients with DR confirming the role of this lnc-RNA as a potential biomarker for DR. MALAT1 was shown to regulate retinal endothelial cell function and microvascular growth in diabetic patients. Liu and colleagues observed that the knockdown of MALAT1 improves DR in vivo and regulates the proliferation of endothelial cells in vitro through the p38-mitogen-activated protein kinase pathway [50]. Furthermore, Zhang and colleagues found an increased secretion of VEGF up-regulating MALAT1, confirming the influence of this lnc-RNA on the regulation of the angiogenetic process [51]. Further molecular studies may aid in the clarification of the exact roles of these lncRNAs in T2DR.

In 2019, Zha and colleagues published the results of their investigation regarding the role of long intergenic non-protein coding RNA p53 induced transcript (LINC-PINT), lnc-RNA known to be involved in tumour cell invasion in human cancers, in T2D retinopathy [44]. The authors analysed the expression of LINC-PINT in 244 T2D patients with different chronic diabetes-related complications (nephropathy, retinopathy, cardiomyopathy, diabetic foot) and 126 healthy subjects, and in ARPE-19 and AC16 cells. LINC-PINT was downregulated in patients with retinopathy, cardiomyopathy or both. The in vitro experiments showed that the treatment with high glucose limited the LINC-PINT expression in the ARPE-19 and AC16 cells, while the overexpression of LINC-PINT increased the viability of ARPE-19 and AC16 cells. Instead, the siRNA-mediated silencing of LINC-PINT elicited the opposite effect. Although further confirmations are necessary, these results suggest a role of LINC-PINT in inhibiting the progression of both retinopathy and cardiomyopathy in T2D patients.

No correlation with T2DR was found for circulating levels of cancer susceptibility candidate 2 (CASC2), growth arrest-specific transcript 5 (GAS5), and RNAs H19 [46,47].

### 3.4. DNA Methylation

Both global and gene-specific DNA methylation are involved in the epigenetic regulation of gene transcription and expression by modulating binding factor or promoting the binding of methyl binding proteins [52]. Emerging evidence suggests a relationship between a hyperglycaemic environment and changes in DNA methylation, identifying these mechanisms as a possible biomarker of diabetic complications [53]. However, the association between DNA methylation and T2DR has been poorly explored (Table 3).

To the best of our knowledge, Maghbooli and colleagues were the first suggesting that differences of the global DNA methylation profile in T2D patients with or without DR could be predictive of this complication [53]. Patients with DR compared to those not affected by DR had a significantly higher content of 5-methylcytosine, assessed to evaluate the global DNA methylation, also after correction for dyslipidaemia, hypertension, hyperglycaemia and duration of diabetes. Moreover, the authors observed a significant increasing trend of global DNA methylation in parallel with DR progression (no DR, 4.22  ±  0.13; NPDR, 4.62  ±  0.17; PDR, 5.07  ±  0.21, *p* = 0.006).

The methylation of the of 5,10-methylenetetrahydrofolate reductase (*MTHFR*) gene promoter has been studied in relation to the onset of DR [54,55]. *MTHFR* is involved in the methionine–homocysteine cycle. The methyl group bound to a cytosine that precedes a guanine can be methylated by *MTHFR* to produce 5-methyltetrahydrofolate which produces methyl donor for the conversion of homocysteine to methionine. Several studies have observed a correlation between *MTHFR* gene polymorphisms and the risk of developing cancer, vascular diseases, diabetes and its complications [57,58]. Dos Santos Nunes and colleagues investigated the methylation profile of *MTHFR* gene promoter and its relationship with biochemical (glycemia, glycated haemoglobin, and lipid level), inflammatory (C-reactive protein and alpha-1 acid glycoprotein) and oxidative stress markers (total antioxidant and malonaldehyde) in Brazilian T2D patients affected or not by DR [56]. They found a significant association between the hypermethylation of the *MTHFR* gene promoter, DR, higher total cholesterol and LDL levels. In 2019, the same group confirmed that the hypermethylated *MTFHR* gene profile, associated with the 1298AA polymorphism of this gene, was related to higher values of glycaemia, total and LDL cholesterol in T2D patients [55]. These results suggest that changes in the methylation of *MTHFR* gene promoter are involved in T2DR onset influencing both homocysteine and lipid metabolism. Dos Santos Nunes and colleagues also studied the methylation profiles of specific miRNA gene promoter in T2D patients [56]. They found that the methylated profile of miR-9-3 was related to an increased risk for DR, while methylated miR-137 could be protective from microvascular diabetes complications. These data showed that the methylation in miRNA promoters may differently affect the course of DR.

## 4. Discussion

DR is a highly specific microvascular complication of diabetes and its main risk factors are long diabetes duration and poor glucose, lipid, and blood pressure control [59,60,61]. In addition, unhealthy lifestyles—low physical activity level, unbalanced diet and tobacco consumption—could directly contribute to the development of DR and other diabetes-related vascular complications [7,62,63]. Nevertheless, it has also been observed that some diabetic patients without these traditional risk factors for DR can likewise develop this complication, suggesting the involvement of other, less well-known, pathogenetic elements [61,64,65]. Thus, over the last decade, researchers have increasingly directed their efforts towards understanding how epigenetics contributes to the initiation and progression of DR. DNA methylation, histone modifications, and miRNAs and lnc-RNA dysregulation have been studied to be proposed for predicting the course of DR in both T1D and T2D. In vitro, in vivo and clinical studies, ruled out in patients with diabetes, have been performed to explore how epigenetic dysregulation contributes, by regulating molecular pathways, to the pathogenesis of diabetes-related microvascular complications. Our study provides a systematic review of epidemiological studies investigating the predictive value of epigenetic biomarkers in T2DR. Most of this research analysed the role of several miRNAs in patients with T2D (Table 1). The expression of miRNAs in human tissues changes as a result of physio-pathological responses. Moreover, due to the properties of stability in biological samples, miRNAs are potentially useful as disease biomarkers [66]. This systematic review summarised the different possible roles of miRNAs in the development of T2DR (Table 1). While the dysregulation of some specific miRNAs (miR-15a, miR-93, miR-93-5p, miR-126, miR-150-5p, miR-184, hsa-let-7a-5p, hsa-miR-novel-chr5_15976, hsa-miR-28-3p) was only related with a higher risk to develop T2DR, for other miRNAs (miR-20b, miR-21, miR-29b, miR-122, miR-155, miR-221, miR-423) an association with the grade of DR was also identified (Table 1). Moreover, the identification of a direct relation between the dysregulation of miR-21, miR-93, and miR-221 and glycometabolic parameters, such as FPG, HbA1c, and HOMA-IR, confirms the crucial role of glucose control and insulin resistance in the development of T2DR, by providing an epigenetic explication of this mechanism [15,17,21,67]. Nevertheless, despite the broad number of miRNAs that have been investigated in relation to T2DR, their application in clinical practice is not currently feasible. Indeed, the majority of the reported results is not reproduced in multiple independent cohorts of T2D patients or, in some cases, conflicting and not univocal. Only in the case of miR-93, miR-126, and miR-221 dysregulation has the evidence obtained from T2D patients been confirmed in cohorts of T1D patients [15,16,17,18,19,20]. To overcome these critical issues, and also due to the heterogeneity of the biological samples and laboratory methodologies, a standardisation of both sample collection and analysis methodologies is mandatory [66]. Certainly, the usage of combinations of multiple biomarkers could improve their predictive value in detecting DR; recently, a panel of three miRNAs (hsa-let-7a-5p, hsa-miR-novel-chr5_15976, and hsa-miR-28-3p) presented a good level of sensitivity and specificity (about 90%) for predicting DR and differentiate early-stage NPDR from late-stage PDR [25].

With respect to the role of lnc-RNA and DNA methylation in T2DR, several studies have been conducted (Table 2 and Table 3). Initial, not univocal evidence is available regarding the possible role of the lnc-RNA MALAT1 dysregulation [28,45] and MTHFR gene promoter hypermethylation [54,55] in T2DR.

## 5. Conclusions

Our systematic review described the main epigenetic biomarkers known to be involved in T2DR. Nevertheless, the aim of their application in clinical practice encounters several challenges because of the insufficient level of the evidence available. There is currently an unresolved need to standardise the biological samples and laboratory procedures and to confirm the obtained data in independent cohort. In clinical practice, the early detection of DR is based on instrumental retinal examinations (fundus oculi, fluorangiography, optical coherence tomography), although the timing and modality of these screening procedures are not often correctly applied on the territory. Nevertheless, the possibility of a tele-retinal evaluation, in particularly in this period of the COVID-19 pandemic, could improve patients’ adhesion to screening programs [68,69].

Of course, an integrated approach including different epigenetic biomarkers, possibly matched with patients’ clinical, instrumental and biochemical features, will be useful to identify accurate panels for the prediction of T2DR. Further prospective investigations are necessary to achieve this aim.

## Figures and Tables

**Table 1:** Summary of the selected studies focusing on miRNA profiling in type 2 diabetes retinopathy.

First Author and Year	Origin	Sample	Marker	T2D Groups (*n*)	Control Group (*n*)	Main Results
Zou H.L., 2017 [15]	China	Plasma	miR-93	DR (75), NDR (65)	HC (127)	miR-93 level increased in DR
Liu H.N., 2018 [17]	China	Serum	miR-221	PDR (30), NPDR (34), NDR (37)	HC (33)	miR-221 level progressively increased in NDR, NPDR and PDR
Rezk N.A., 2016 [16]	Egypt	Serum	miR-126	DR (19), NDR (81), IGT (86)	HC (100)	miR-126 level decreased in DR
Jiang Q., 2017 [21]	China	Plasma	miR-21	PDR (51), NPDR (73), NDR (65)	HC (115)	miR-21 level progressively increased in NDR, NPDR and PDR
Kamalden T.A., 2017 [22]	Malaysia	Plasma	miR-15a	PDR (15), NPDR (22), NDR (41)	HC (19)	miR-15a level increased in DR
Blum A., 2019 [23]	Israel	Serum	miR-423	PDR (15), NPDR (22), NDR (10)	HC (22)	miR-423 level progressively decreased in NDR, NPDR and PDR
Dantas da Costa E Silva M.E., 2019 [24]	Brazil	Plasma	miR-29b miR-200b	PDR (49), NPDR (46), NDR (91)	HC (20)	miR-29b and miR-200b level progressively decreased in NDR, NPDR and PDR
Liang Z., 2018 [25]	China	Serum	hsa-let-7a-5p hsa-miR-novel-chr5_15976 hsa-miR-28-3p	DR (29), NDR (50)	None	hsa-let-7a-5p and hsa-miR-28-3p level increased in DR hsa-miR-novel-chr5_15976 level decreased in DR
Yang T.T., 2015 [26]	China	Serum	miR-155	PDR (20), NPDR (20), NDR (18)	HC (20)	miR-155 level progressively increased in NDR, NPDR and PDR
Pastukh N., 2019 [27]	Israel	Serum	miR-122	PDR (10), NPDR (10), NDR (10)	HC (10)	miR-122 level progressively increased in NDR, NPDR and PDR
Shaker O.G., 2019 [28]	Egypt	Serum	miR-20b	PDR (20), NPDR (30), NDR (30)	HC (81)	miR-20b level progressively decreased in NDR, NPDR and PDR
Ma J., 2017 [29]	China	Serum	miR-3939 miR-1910-3p	DR (5), NDR (5)	None	No differences of miR-3939 miR-1910-3p level
Rovira-Llopis S., 2018 [30]	Spain	Serum	miR-31	DR (13), NDR (31)	HC (24)	No differences of miR-31 level
Chen S., 2019 [31]	China	Aqueous humour	miR-93-5p miR-184 miR-150-5p	PDR (9)	HC (9)	miR-93-5p, miR-184, and miR-150-5p level decreased in PDR
Pinazo-Duran M.D., 2016 [32]	Spain Portugal	Tears	Panel of 14 miRNAs	DR, NDR (77)	HC (55)	miRNAs level increased in DR and NDR groups

Abbreviations: DR, diabetic retinopathy; NDR, not diabetic retinopathy; HC, healthy controls; PDR, proliferative diabetic retinopathy; NPDR, non-proliferative diabetic retinopathy; IGT, impaired glucose tolerance.

**Table 2:** Summary of the selected studies focusing on long non-coding RNA profiling in type 2 diabetes retinopathy.

First Author and Year	Origin	Sample	Marker	T2D Groups (*n*)	Control Group (*n*)	Main Results
Shaker O.G., 2019 [28]	Egypt	Serum	MALAT1 HOTAIR	PDR (20), NPDR (30), NDR (30)	HC (81)	MALAT1 and HOTAIR level increased in NPDR and PDR
Toraih E.A., 2019 [45]	Egypt	Plasma	MALAT1 RNCR2	DR (75), NDR (55)	HC (108)	MALAT1 and RNCR2 level decreased in DR
Zha T., 2019 [44]	China	Plasma	LINC-PINT	DR, NDR (244)	HC (126)	LINC-PINT decreased in DR
Wang L., 2018 [46]	China	Serum	CASC2	DR (33), NDR (146)	HC (56)	No differences of CASC2 level
Fawzy M.S., 2020 [47]	Egypt	Plasma	H19 GAS5	DR (66), NDR (53)	HC (110)	No differences of lnc-RNA H19 and GAS5 level

Abbreviations: MALAT1, metastasis-associated lung adenocarcinoma transcript 1; HOTAIR, homebox antisense intergenic RNA; RNCR2, retinal non-coding RNA2; PDR, proliferative diabetic retinopathy; NPDR, non-proliferative diabetic retinopathy; NDR, not diabetic retinopathy; HC, healthy controls; DR, diabetic retinopathy; LINC-PINT, long intergenic non-protein coding RNA p53 induced transcript; CASC2, cancer susceptibility candidate 2.

**Table 3:** Summary of the selected studies focusing on global and gene-specific DNA methylation in type 2 diabetes retinopathy.

First Author and Year	Origin	Sample	Marker	T2D Groups (*n*)	Control Group (*n*)	Main Results
Maghbooli Z., 2015 [53]	Iran	PBL	Global DNA methylation (5-methylcytosine content)	PDR, NPDR (74) NDR (94)	None	Global DNA methylation progressively increased in NDR, NPDR and PDR
Dos Santos Nunes M.K., 2017 [54]	Brazil	PBL	Methylation of *MTHFR* gene promoter	DR (16), DN (29)	T2D with no complications (60)	*MTHFR* gene promoter hypermethylation is associated with DR
Santana Bezerra H., 2019 [55]	Brazil	PBL	- Methylation of *MTHFR* gene promoter; - Polymorphisms C677T and A1298C of *MTHFR* gene	DR (22), NDR (25)	T2D with no complications (60)	*MTHFR* gene promoter hypermethylation associated with the 1298AA polymorphism was related to higher values of glycaemia, total cholesterol and LDL cholesterol
Dos Santos Nunes M.K., 2018 [56]	Brazil	PBL	Methylation of miR-9-3, miR-34a, and miR-137 gene promoter	DR (19), DN (29)	T2D with no complications (60)	Hypermethylation of miR-9-3 gene promoter was related to an increased risk for DR, while hypermethylation of miR-137 gene promoter could be protective from microvascular diabetes complications.

Abbreviations: PBL, peripheral blood leucocytes; PDR, proliferative diabetic retinopathy; NPDR, non-proliferative diabetic retinopathy; NDR, not diabetic retinopathy; DR, diabetic retinopathy; DN, diabetic nephropathy; T2D, type 2 diabetes.

## Data Availability

Data used to support the findings of this study are available from the corresponding author upon request.
